# A synthesis of women’s participation in small-scale fisheries management: why women’s voices matter

**DOI:** 10.1007/s11160-023-09806-2

**Published:** 2023-10-18

**Authors:** Mouna Chambon, Sara Miñarro, Santiago Alvarez Fernandez, Vincent Porcher, Victoria Reyes-Garcia, Huran Tonalli Drouet, Patrizia Ziveri

**Affiliations:** 1 Institute of Environmental Science and Technology, Universitat Autònoma de Barcelona, (ICTA-UAB), 08193 Barcelona, Bellaterra Spain; 2 Institut de Recherche Pour Le Développement (IRD) & Centre de Coopération Internationale en Recherche Agronomique Pour Le Développement (Cirad), Unité Mixte de Recherche “Savoirs Environnement Sociétés”(SENS), Montpellier, France; 3 Institució Catalana de Recerca I Estudis Avançats (ICREA), 08010 Barcelona, Spain; 4 Department d’Antropologia Social I Cultural, Universitat Autònoma de Barcelona, 08193 Barcelona, Bellaterra Spain; 5 Université Libre de Bruxelles, Avenue Franklin Roosevelt 50, 1050 Brussels, Belgium; 6 Department de Biologia Animal, Biologia Vegetal I Ecologia, Universitat Autònoma de Barcelona, 08193 Barcelona, Bellaterra Spain

**Keywords:** Artisanal fisheries, Fisheries management, Gender, Inland fisheries, Ocean sustainability, Women

## Abstract

While women globally make up nearly half of the fisheries workforce, their contribution to the sector has long been overlooked with implications for fisheries management. To assess women’s participation in small-scale fisheries (SSF) management and related socio-cultural, environmental, and economic impacts, we conducted a systematic review of peer-reviewed literature (n = 124 case studies). Women had no or limited participation in more than 80% of the examined case studies reporting their participation level in SSF management. Women’s exclusion from SSF management resulted in negative outcomes, whereas their active participation was associated with various positive impacts at multiple scales. Most of the documented impacts were socio-cultural, suggesting a gap in documenting environmental impacts stemmed from women’s participation in SSF management. Importantly, most impacts reported affected the social-ecological system scale, suggesting that gender inclusion may contribute to improving the management of SSF social-ecological systems. We conclude by highlighting the need to foster gender perspectives in data collection methods used in fisheries research, in SSF management, and in ecological research on SSF social-ecological systems.

**Supplementary Information:**

The online version contains supplementary material available at 10.1007/s11160-023-09806-2.

## Introduction

Despite the entrenched view that fishing is a male domain (Lentisco & Lee 2015), women actually make up 47% of the fisheries workforce worldwide (FAO 2016). Women’s contribution to small-scale fisheries (SSF), also called subsistence or artisanal fisheries, is particularly important given that this sector represents a key source of protein for millions of people globally, especially in coastal communities (Österblom et al. 2020). Although women engage in different parts of the SSF value chain, their participation in the sector has long been invisible, ignored, and unrecognized (Harper et al. 2013; WSI 2020), partly due to narrow definitions of fishers, which exclude women’s fishing activities, and partly due to gender-biased sampling methods (Kleiber et al. 2015). In El Salvador, for instance, fishers are defined as people capturing fish in the open sea using boats and nets. Since Salvadorian fisherwomen seldomly use a boat to fish, their contribution to the SSF economy remains unreported in national fishery statistics (Gammage 2004). This definition focusing on the capture node of the SSF value chain also makes invisible Salvadorian women’s work in pre- and post- production activities. Data collection methods in fisheries research may also lead to the under-representation of the number of women engaging in fisheries activities. This is the case for example of survey methods targeting household heads, which may favour male over female respondents (Kleiber et al. 2015).

Against this background, over the last 30 years, some scholars have strived to give visibility to women’s involvement in SSF. This trend started with the “women in fisheries” (WIF) approach, which focused on women’s multiple contributions to the SSF sector (Williams et al. 2002). This research field shed light on the various roles played by women all along the SSF value chain, from pre- to post-production. WIF studies highlighted how women take part in time-consuming pre-production tasks such as repairing nets (Browne 2002; Sotto et al. 2001) as well as in direct fish capture activities, including the case of fisherwomen using scoop nets and traps in Malaysia (Yahaya 2001), or boats to harvest small sardines (“dagaa”) in the Tanzanian side of Lake Victoria (Tungaraza 1986). Several scholars also documented women’s involvement in post-production activities (Ahmed et al. 2001; Siason et al. 2001). Finally, some other studies with the same approach have looked at women’s caring activities for communities and households, which are also essential for the maintenance of fishing activities (dela Pena & Marte 2001; Sotto et al. 2001). More than other activities, women’s care-work, although critical for sustaining SSF activities, is often informal, unpaid, and overlooked (Williams 2019).

More recently, the “gender and fisheries” (GAF) perspective has emerged as a new approach to document the importance of gender –defined as the socially constructed attributes associated with what is to be a female or male in various socio-cultural contexts- in SSF value chains (Bennett 2005; Williams et al. 2002). In that sense, GAF research has explored topics ranging from intersectionality in SSF to feminist fisheries political economy (Williams 2019). As it has become increasingly recognized that women engage in every step of the SSF value chain, GAF research has stressed the need for gender perspectives in SSF management processes (Williams 2010).

Fisheries management refers to the complex and continuous process aiming at using fisheries resources sustainably through different stages, from setting management plans and objectives to the implementation of required actions (Berkes et al. 2001; FAO, 1997). Scholars distinguish three main approaches to SSF management depending on the participation level of resource users: top-down processes, defined by a distant central government with no or little participation of local communities; community-led management, or approaches in which all resource users are directly and fully participating in natural resource management; and co-management, characterized by a shared authority between the community and the central government for managing fishery resources (Twyman 2017). While there are various definitions of participation, in this study we adopt the one proposed by Agarwal (2001) who broadly defines participation as “a dynamic interactive process in which the disadvantaged have voice and influence in decision-making” (Pag:1624). Some scholars have criticized such concept suggesting that participatory approaches are more likely to reinforce existing social inequalities instead of shifting power relationships (Cooke & Kothari 2001; Stone & Nyaupane 2014). Conversely, a wide range of empirical studies have documented the benefits of participation for conservation outcomes and equity (Gilmour 2016; Akhtaruzzaman et al. 2023). In the SSF sector, local communities’ participation in fisheries management has increasingly been promoted by institutions (FAO 2015) and researchers (Cohen & Steenbergen 2015; Jentoft 2005; Jupiter et al. 2014), gradually shifting the debate from a focus on the participatory approach itself to its implementation on the ground (Berkes & Nayak 2018).

In this context, a growing body of literature has emphasized the need to increase women’s participation in fisheries management both for intrinsic (i.e., for its own sake) and instrumental motivations (i.e., as a mean to achieve specific outcomes) which, in turns, influences the ways that gender equity is assessed in practice (Lawless et al. 2021). On the one hand, achieving gender equity in SSF management is seen as desirable for fairness and justice principles, to guarantee that both women and men have the same rights and opportunities (FAO 2016). On the other hand, women’s participation in SSF management has also been promoted as a mean to prevent socio-environmental pitfalls (Kleiber et al. 2013; Rohe et al. 2018). In particular, previous studies have documented how failing to incorporate gender considerations in SSF management may lead to an overall under-evaluation of the actual fishing effort and catches (Mills et al. 2011), biased understandings of coastal social-ecological systems (de la Torre-Castro 2019; Kleiber et al. 2015) or negative social impacts (Weeratunge et al. 2010; Williams 2010).

Facing these concerns, international agencies (e.g., FAO 2017) and researchers (e.g., de la Torre-Castro 2019; Koralagama et al. 2017) have called for the adoption of inclusive management processes, including all resource-users, regardless of their gender, and recognizing their knowledge, perspectives, and needs in fisheries management (de la Torre-Castro 2019; Nessa et al. 2020; Resurreccion 2006). On the institutional front, the FAO created in 2017 a dedicated handbook on gender equity in SSF as an extension of the SSF Guidelines (FAO 2015) to specifically address SSF challenges while improving gender equity in SSF management. In academia, research on the topic has significantly grown over the past decade (Galappaththi et al. 2022) particularly focusing on women’s tasks in SSF governance (Galappaththi et al. 2022) and barriers and enablers for women’s participation in SSF management and governance (Galappaththi et al. 2022; Lentisco & Lee 2015). Some scholars have also explored the impacts related to the participation of women in such arenas. Yet, the few studies on the topic have used a limited sample size for the SSF sector (Galappaththi et al. 2022; Lentisco & Lee 2015) or did not find sufficient evidence to be extrapolated at the global level (Leisher et al. 2016, 2017). To the best of our knowledge, no studies systematically assessed the extent of women’s participation in SSF management and related impacts at a global scale and using a large sample size. This gap is surprising, as researchers have documented impacts associated to the engagement of women in other sectors including ecological restoration (Broeckhoven & Cliquet 2015), forestry conservation (Agarwal 2009), and environmental management (de la Torre-Castro 2019). Addressing this research gap is not only necessary for intrinsic reasons, from a human rights perspective, but also to favour effectiveness in the sustainable use of fisheries resources in line with the FAO’s SSF guidelines (2015), the Convention on Biological Diversity’s Global Biodiversity Framework (target n°23) and the Sustainable Development Goals Indicators framework (SDG n° 5 and 14 in particular).

To address this knowledge gap, our work reviews documented impacts associated to strengthening women’s participation in SSF management and decision-making. The originality of our review is to provide the first global quantitative assessment of women’s participation level in SSF management and related socio-cultural, environmental, and economic impacts. We acknowledge the heterogeneity and diversity that exists amongst women and, more broadly, people of diverse gender identities in consideration with intersectional aspects (Kenny & Tapu-Qiliho 2022). However, for the purpose of our review, we chose to focus on women as a group of study since much of the literature on gender and fisheries applies a binary view and tends to emphasize women’s roles within the sector as different from men’s roles (House et al. 2023). Specifically, we ask the four following questions to the existing peer-reviewed literature:

1. What is the extent of women’s participation in SSF management?
2. What are the socio-cultural, environmental, and economic impacts associated to women’s participation -or lack thereof- in SSF management?
3. How does the direction of these impacts (i.e., positive, negative) vary depending on women’s participation level in SSF management?
4. At what scale are these impacts unfolding (i.e., individual, community, SES (socio-ecological system))?

## Methods

### Publication selection

**Fig. 1:**
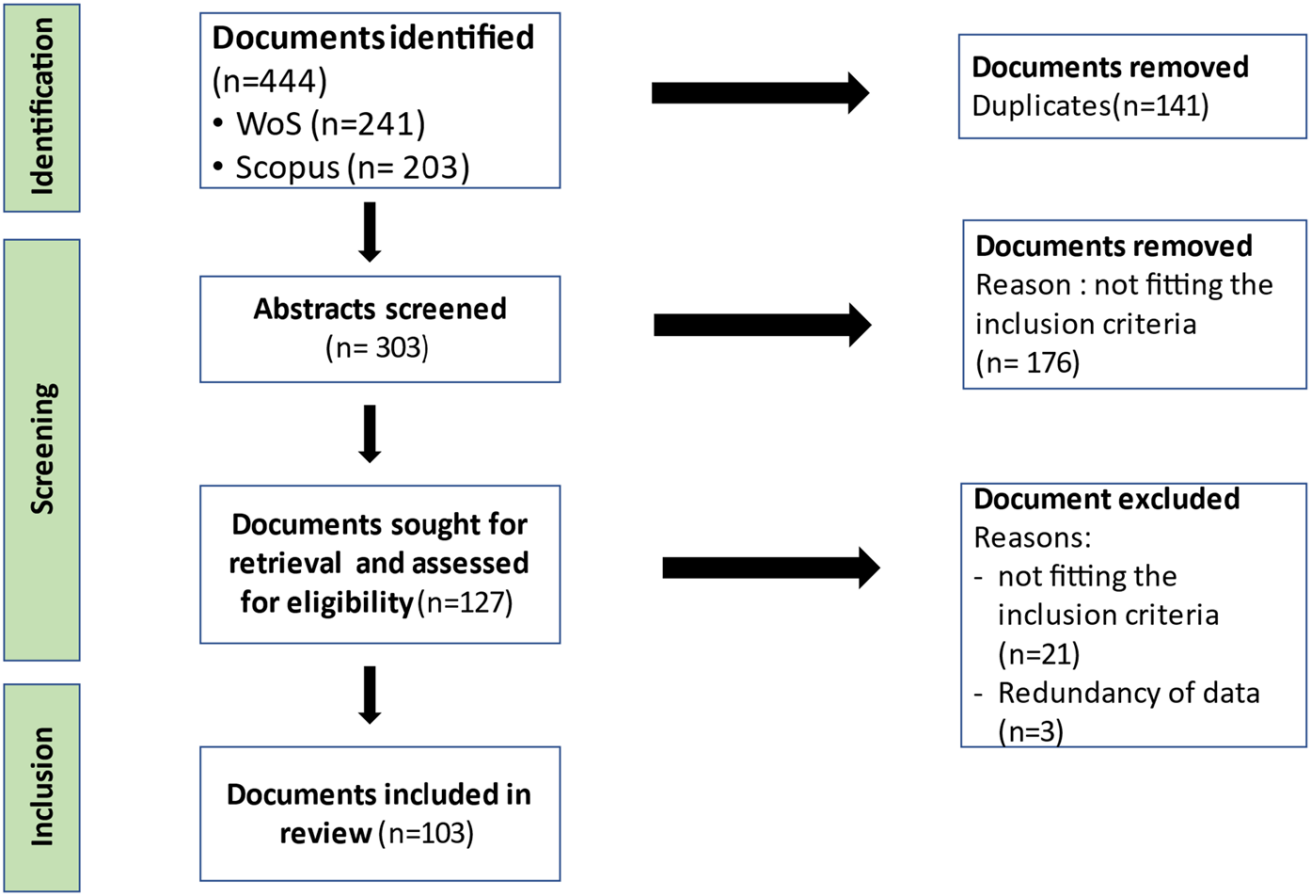
Flow chart presenting the selection of documents

We built on the methodological principles of the systematic literature review (Haddaway et al. 2015) to synthesize the existing evidence on women’s participation in SSF management and related impacts in peer-reviewed journal articles, book chapters, and conference papers. To assess the literature, we performed a topical search (in title, abstract, and keywords) in two databases: Web of Science (WoS) Core Collection and Scopus. We conducted an initial search in June 2021 and updated it in June 2022. To select our search terms, we drew on Smith and Basurto’s (2019) review which defines specific keywords for SSF that cover both current (e.g., artisanal fisher, Aburto et al. 2021) and historical terms (e.g., small-scale fisher, Thomson 1980) referring to SSF. We adjusted these search terms to include gleaning activities which are often female-dominated (de la Torre-Castro 2019) and added key words related to gender and management. The search string used was TOPIC: “small-scale fish*” OR “local fish*” OR “traditional fish*” OR “artisanal fish*” OR “subsistence fish*” OR “glean* “AND TOPIC: “gender*” OR “women* “AND TOPIC: “Management” WITHOUT TOPIC: “sex ratio”. We used asterisks to broaden the scope of the search outputs. Our search did not include any geographical restriction and included both inland and marine fisheries. The search resulted in a total of 444 entries (WoS = 241; Scopus = 203), from which we removed 141 duplicates to screen a total of 303 publications (Fig. 1).

For a publication to be reviewed, it had to meet four inclusion criteria: (i) use primary data, (ii) have an explicit SSF context, (iii) explicitly mention the term fisheries management in the body of the article (either to describe management processes and activities in the local SSF context, or to provide further recommendations) and (iv) include information on women’s participation in at least one stage of the SSF value chain (i.e., pre-production, production, post-production, care-work) and/or management tasks and activities (e.g., monitoring, administration). We chose these inclusion criteria to capture women’s participation in SSF management in its broadest sense, recognizing the complexity, diversity and tangled nature of the SSF sector (Smith and Basurto 2019). Decisions on inclusion were taken during the screening process which followed two stages. First, two coders simultaneously screened the titles and abstracts considering the inclusion criteria. Second, the lead author performed the full-text screening for all publications that passed the first selection stage. In total, we identified 103 peer-reviewed publications detailing women’s participation in SSF management and related impacts. The list of publications included and the justification for document’s exclusion is presented in the supplementary material (Online Resources 1 & 2).

### Data collection and coding

**Fig. 2:**
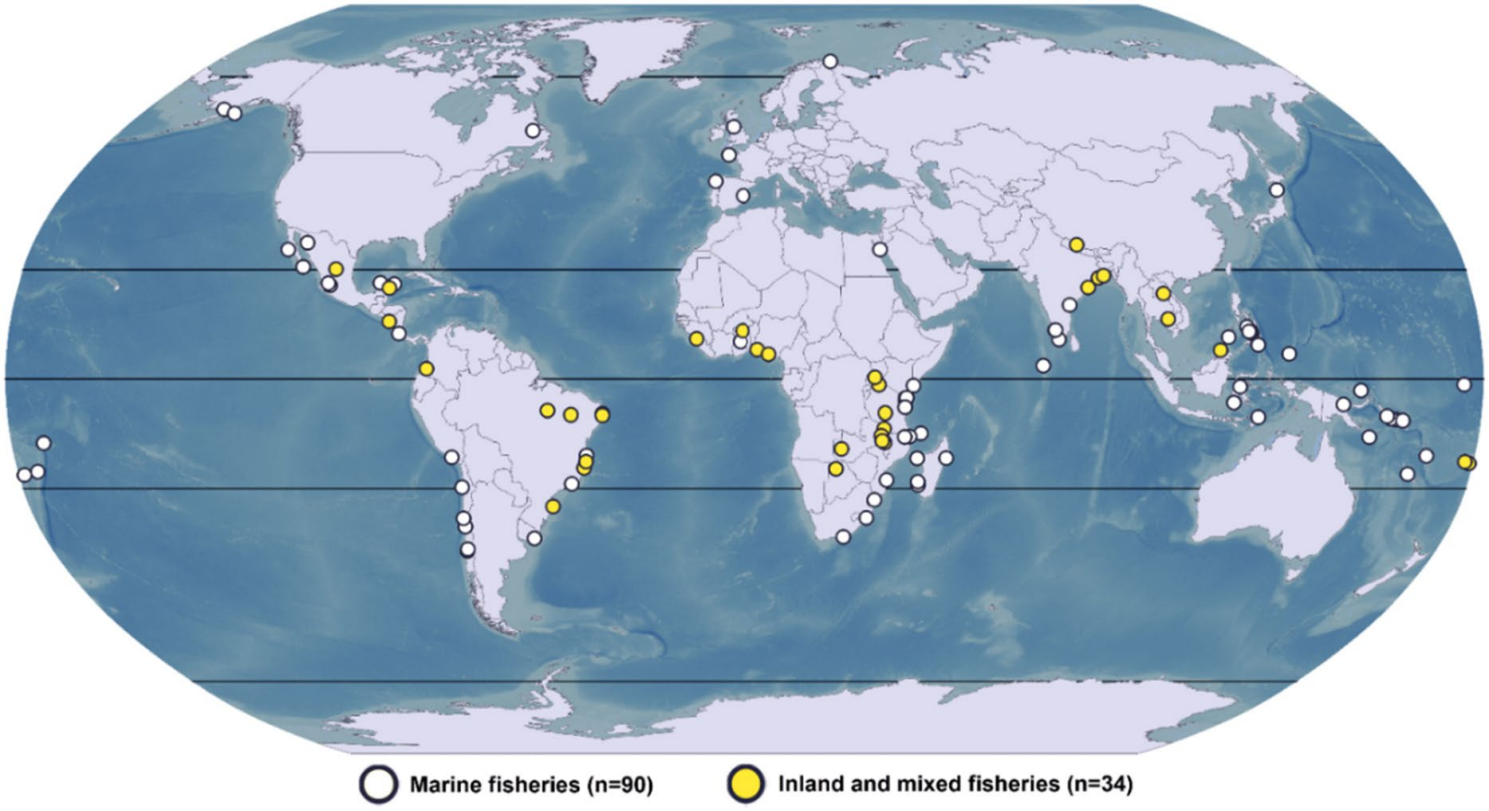
Geographical location of the 124 case studies, per fisheries type. The map was built under QGIS 3.22.7, using bathymetric data from General Bathymetric Chart of the Oceans (gebco.net)

We collected data from the selected publications and coded information regarding women’s participation in SSF management and related impacts, defined here as outcomes affecting the socio-cultural, environmental, or economic dimension of individuals, communities, or SES (Online Resource 3). Two coders read the publications and coded the information following a two-step process. First, each coder was allocated half of the publications to read and code. In a second stage, the lead author checked the quality of data entry and verified the uniqueness of each publication to avoid double counting those based on the same case study. Some publications reported information from more than one case study. In these cases, we collected information separately for each case study, resulting in a total sample size of 124 case studies (Fig. 2).

For each publication, we recorded *verbatim* statements from selected publications referring to women’s participation in SSF management. We then classified women’s participation levels in SSF management. To do so, we simplified Agarwal’s typology (2001) of women’s participation in natural resource management by using three main level categories: excluded, limited participation, and active (Online Resource 4). Then, we examined the reported impacts of women’s participation in SSF management by categorizing the reported impacts as socio-cultural, environmental, or economic. Within this broad division, we created 18 subcategories of impacts using an inductive qualitative content analysis. To design these subcategories, we read through the *verbatim* statement of impacts extracted from the publications and applied in vivo coding to identify emerging types. This qualitative method is useful for coding data with a strong emphasis on *literatim* statements (Manning 2017). In addition, we reported the direction of each impact using the same extracted *verbatim* statements and based on how the impact was framed by the author, either as a positive or negative impact. Finally, we differentiated between three scales for each impact: the entire SES, the community -including households-, and the individual scales.

### Data analysis

To assess the extent of women’s participation in SSF management (RQ1) and explore related impacts (RQ2), we used descriptive statistics. Specifically, we counted the frequency of each reported women’s participation level in SSF management as excluded, limited participation, and active. Likewise, we ordered the different categories of impacts (i.e., socio-cultural, environmental, and economic) by frequency count. Where relevant, we illustrated quantitative results with quotes from the documents examined. To examine the relation between women’s participation level in SSF management and impact direction (RQ3), we first distinguished between impacts related to either the exclusion or participation (i.e., limited; active) of women in SSF management, as well as their direction (i.e., positive or negative). Then, taking the case study as unit of analysis, we used the Pearson’s Chi-squared test (Pearson 1900) with Yates’ continuity correction to statistically assess the relationship between women’s participation level and impact direction. To provide a visual representation of this relationship, we used a non − metric multidimensional scaling (NMDS) as a rank-based approach that spatially displays the distance between objects in a low-dimensional space. This method can be used both with qualitative and quantitative variables (Kruskal 1964). All statistical analyses were performed with R Statistical Software (v4.2.1; R Core Team 2021) using the *vegan* package (Oksanen et al. 2022). We considered that a difference was statistically significant when p-values were below 0.05. Finally, to assess the scale of the reported impacts (RQ4), we used descriptive statistics to count the frequency distribution of each reported impact unfolding either at the SES, community or individual scales and combined this analysis with quotations.

## Results

### Women’s participation in SSF management

Among the 124 case studies documented, 75 (60%) provided information on women’s participation in SSF management. The other case studies did not give details specific enough to categorize women’s participation. In these cases, the authors often provided insufficient information on gendered differences in relation to SSF management or did not mention women’s role in these processes. Furthermore, we found geographical variations in gender-data provided in the reviewed literature. While most case studies were in Africa (n = 36), Oceania (n = 23) and Asia (n = 22), we found that only about 50% of the cases in Africa and Asia reported on women’s participation level in SSF management. Conversely, although we recorded fewer studies in Europe (n = 6), all of them provided this information.

Within the subsample of studies detailing women’s participation level in SSF management, more than 80% of the case studies reported either women’s limited participation or exclusion from SSF management. In most cases, women had limited participation in SSF management (n = 39), with the documents typically signalling that women attended management meetings but did not have full opportunities to speak up and influence the outcome. This situation is illustrated by Singleton’s et al. (2019) study on SSF communities in Southwest Madagascar: “Whilst women may attend meetings, few voice opinions at them, and few are confident that they have influence (…)” (p. 8). Common barriers that hampered women to actively participate in SSF management processes included cultural norms and gender stereotypes that contribute to undervalue women’s opinions in meetings and alter their confidence. For instance, women’s limited participation, as reported by Singleton et al. (2019), was directly associated to their sense of inferiority regarding old Malagasy men who also attended SSF management meetings in their communities: “In follow-up focus groups, women stated (…) their opinion is not respected in the presence of ‘*nahodas*’ (older men)” (p. 8).

Nearly a third of the examined cases with information on women’s participation in SSF management (n = 22) reported the exclusion of women from management processes. In those cases, women were not joining SSF management meetings, nor other related tasks or events, either owing to formal exclusion -such as not being a member of the local SSF management body- or to informal social barriers. For example, Cele (2020) describes how female mussel collectors in poor black communities in coastal South Africa face formal gender barriers to access to SSF management activities: “(…) even when State departments such as DAFF [Department of Agriculture, Forestry and Fisheries], and Ezemvelo KZN Wildlife engage with community-based fishing organisations, they do not invite women mussel harvesters to these meetings. Such tendencies deprive women of access to pertinent harvesting information, and training, and further perpetuate gender divisions in the industry” (p. 145). Entrenched gender inequalities can also prevent women to join these activities, even if their formal access is guaranteed. In particular, domestic duties and childcare were reported as a major constraint for women as SSF management meetings often happen in the evening, thus overlapping with women’s house chores and excluding them de facto from these events (Gustavsson et al. 2021; Santos 2015; Torell et al. 2021).

By contrast, only 14 case studies documented women’s active participation in SSF management. This is, for example, the story of the CoopeTarcoles R.L SSF cooperative in Costa Rica: “Slowly, CoopeTárcoles R.L has been expanding beliefs on the role of women, promoting the fact women can and do play an active role and contribute on a daily basis to the community’s economic, social, and cultural life. Women have been accepted in the cooperative and have taken active roles in the Administrative Council” (Rivera et al. 2017, p.13).

### Typology of impacts related to women’s participation in SSF management

Among the 124 selected case studies, 98 (about 80%) reported impacts related to women’s participation in SSF management. We recorded a total of 190 socio-cultural, environmental, and economic impacts derived from either women’s participation (n = 121) or exclusion (n = 69) in SSF management. We documented impacts considering 18 different categories as presented in Table 1.

**Table 1:** Reported socio-cultural, environmental, and economic impacts related to women’s participation- or lack of – in SSF management processes

Scale	Category	Subcategory	Example	Corresponding quote & reference
Social-ecological system	Socio-cultural	Change in the understanding of the gender dynamics within the SSF^a^ SES^b^ (Comprehensiveness)	Gendered perspectives in SSF management in Brazilian Amazonia highlight differences between fishermen and fisherwomen’s practices, hence providing a comprehensive view for fish stock assessments	“*The study results also confirmed our hypothesis that differences between fishermen and fisherwomen in species composition of catches were influenced by the types of gear used and fishing sites explored.”* (Zacarkim et al. 2015, p. 415)
		Change in the impact of management decisions on women (Gendered management impact)	Women’s exclusion from the designation process of a marine closure in the Solomon Islands has negative social consequences on their daily lives	*“Women were more constrained in their fishing activities because a marine closure was located where mainly women used to fish. Our study highlights the importance of paying attention to women’s needs and actions in the governance of the fishery.”* (Rohe et al. 2018, p.155)
		Change in the recognition of gendered ecological knowledge (Gendered ecological knowledge)	The deep-rooted knowledge of fisherwomen on salmon distribution and abundance can guide the management of salmon fisheries in Alaska	*“The experience and knowledge of these women can inform fishery managers of various aspects of environmental change. For example, their knowledge of change in salmon distribution and abundance over the years, can be used to triangulate data used by managers for decision making regarding the resource.” (*Lavoie et al. 2019, p.336)
		Change in the compliance to management measures (Compliance)	The engagement of women’s and men’s groups contribute to fostering local support and acceptance to a fishery management plan in Samoa	*“Regardless of legislation or enforcement, the responsible management of marine resources will only be achieved when fishing communities themselves see it as their responsibility. Accordingly, the strategy focused on mobilizing each community through direct contact with key village groups. These included women’s groups and untitled men’s groups to ensure the widest community participation and eventual ownership of the village fisheries*.” (King & Faasili 1999, p.2)
		Change in the diversity of perspectives for SSF management (Diverse perspectives)	Women’s participation in SSF management leadership positions brings new views and skills that favour the development of co-management in the Chile’s Biobio region	“*Some of them* [fishermen] *mentioned that women’s management skills when assuming a leadership position was favored: “In a short time, she has gotten two projects that never happened before... women have another way of thinking (fisherman member #9).”* (Franco-Melendez et al. 2021, p.14)
	Environmental	Change in the long-term use of fisheries resources (Sustainable management)	The participation of Galician women in the management of shellfish in Spain allows to sustain shellfish resources over the long term	*“Some interviewees highlighted the particular management logic of the shell fisherwomen, in which they take a systemic view of resource* *Management.”* (Fadigas 2017, p.565)
		Change in human pressure on local ecosystems (Ecological pressure)	The exclusion of Fijian fisherwomen from fisheries management decisions raises concerns about the risk of increasing ecological pressure on coastal resources	*“As many more women enter commercial markets, there is a growing concern that women may be harvesting and selling undersized juvenile fish from these habitats, affecting the sustainability of some of the common fisheries.”* (Thomas et al. 2021, p.7)
Community	Socio-cultural	Change in food security (Food security)	The important role of fisherwomen for providing fresh fish to their households has implications for fishery management in the Maldives	*“This suggests that women’s involvement in small-scale reef fisheries, while not necessarily direct, might still be important to consider especially from the point of view of island food systems and the processes that contribute to the nutritional health of communities.”* **(**Yadav et al. 2021 p.3)
		Change in adaptive capacity (Adaptive capacity)	Women’s roles in small-scale fishing communities in Peru and Japan contribute to improve the capacity of their community to adapt to external chocks	“*In the two cases reported here, women are taking responsibilities and applying innovative activities to adapt to disturbances in the fishery system*.” (Delaney et al. 2019, p.292)
		Change in the social attributes of the community (Community social attributes)	Women’s action through self-help groups favours solidarity within small-scale fishing communities in Kerala, India	“*The empowerment of women through SHGs leads to benefit not only the individual women and women groups, but also the family and community as a whole through collective action and solidarity.*” (Jeeva & Gopal 2021, p.175)
		Change in the transmission of traditional knowledge (Cultural heritage)	The participation of native women in a Marine Extractive Reserve in Brazil has the potential for preserving the traditional knowledge of their community	“*Their participation [of women] in the management is expected to contribute rules for political strengthening and income production, thus keeping the traditional knowledge and maintaining the native population in the area*.” (Di Ciommo 2007, p.65–66)
	Economic	Change in community income (Community income)	Women’s participation in SSF management through fishing permits in Isla Arena, Mexico, brings more revenue for their households	“(…)*an aspect that is cross-sectional in these arrangements is financial motivation, since having the permits is something that strengthens the reception of economic resources by the families.*” (Uc-Espadas et al. 2017, p.392)
Individual	Socio-cultural	Change in well-being (Well-being)	Women’s engagement in the management of their local fisheries resources in Chile’s Biobio region enhances their feelings of tranquillity and security	“*Several fisherwomen emphasised the feelings of tranquillity and the security of having something of “their own” that they could nurture*.” (Gallardo-Fernandez & Saunders 2018, p.184)
		Change in capacity building (Capacity building)	Fisherwomen who are part of a union in Chile’s Biobio region learn new skills in the process of managing their local fisheries resources	*“We learned to work together… in group; because this work was always done individually; we learned to manage; we learned to find nexus networks, in which to support us to continue the struggle.”* (Gallardo-Fernandez & Saunders 2018, p.184)
		Change in women’s empowerment (Empowerment)	Women’s participation in a Costa Rican SSF cooperative gives them personal confidence	*“ (…) opportunities have opened up for some of the women fishing leaders to participate in activities, conferences and seminars that broaden their horizons and build their self-esteem***.”**(Rivera et al. 2017, p.13)
		Change in gender roles (Gender roles)	Women’s participation in the co-management of arapaima fisheries in Brazilian Amazonia contributes to change gender dynamics and alter traditional gender roles	“*They* [Women] *also pointed out the opportunity of having both genders working together, and of women being able to take part in an activity that used to be male-dominated.” (*Freitas et al. 2020, p.6)
		Change in women’s leisure time (Women’s leisure time)	The transference of fishing permits from men to women in Isla Arena, Mexico, is associated with an additional workload that reduces women’s free time	“*We found testimonies such as the following where the woman permit holder expressed that this condition represents a load of tasks for women, particularly because of the paperwork and trips they have to make” (*Uc-Espadas et al. 2017, p.394)
	Economic	Change in women’s income (Women’s income)	Women’s participation in the co-management of arapaima fisheries in Brazilian Amazonia leads to an increase in their fishing revenue	“ (…) *woman living in a community with arapaima co-management would* *have a mean probability of 77% of earning money from fisheries,* *compared to only 8% for a woman living in a community without* *arapaima co-management.*” (Freitas et al. 2020, p.5)

^a^Small-scale fisheries (SSF)

^b^Social-ecological system (SES)

**Fig. 3:**
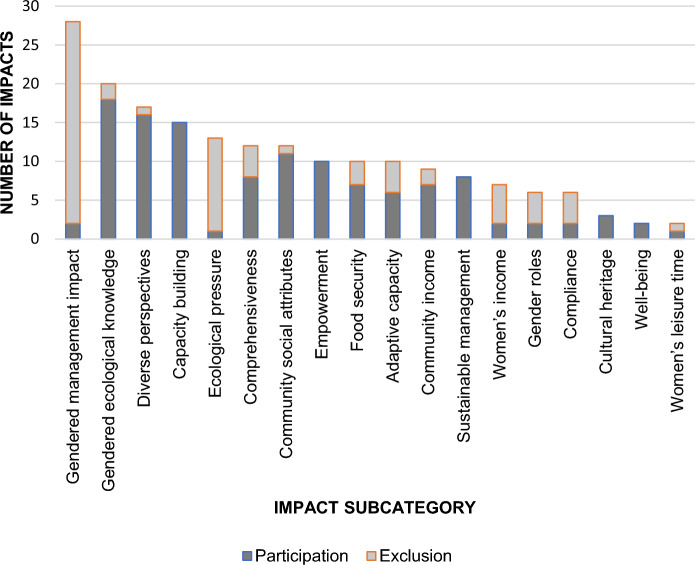
Number of impacts per subcategory (n = 190). The impacts displayed in dark grey are associated to cases where women participate in SSF management (i.e., limited or active participation) whereas impacts in light grey are those associated to women’s exclusion cases

Most reported impacts of women’s participation in SSF management were socio-cultural (n = 153). The most frequently reported impact was change in the impact of management decisions on women (n = 28), referring to cases where the management measures taken had unintended social consequences on women’s lives. In most reviewed cases (93%), this impact was associated to women’s exclusion from SSF management processes rather than their participation (Fig. 3). For instance, women in the Solomon Island were excluded from a discussion on the designation of a marine closure, which had a negative social impact on their daily life: “Women were more constrained in their fishing activities because a marine closure was located where mainly women used to fish” (Rohe et al. 2018, p. 155). Another very common socio-cultural impact was change in the recognition of gendered ecological knowledge (n = 20). This impact was predominantly associated to cases where women participated in SSF management (limited or active participation). In those cases, women’s participation in management processes allowed to express specific gendered local knowledge that helped management operations such as determining salmon stocks (Lavoie et al. 2019), generating a map on fisheries resources (Paul et al. 2016), or assessing SES vulnerability in coastal communities (Tilley et al. 2021). The third most cited impact was change in the diversity of perspectives for SSF management (n = 17). Women’s participation in SSF management was associated to new viewpoints and skills that broadened the scope of reflection for management processes. As an illustration, women’s participation in leadership positions in the Chile’s Biobio region led to the development of a co-management programme owing to their organizational and managerial skills (Franco-Melendez et al. 2021).

Women’s level of participation in SSF management had a much lower number of reported environmental (n = 21) and economic impacts (n = 16). The most common environmental impact associated to women’s participation in SSF management was change in human pressure on local ecosystems (n = 13), whereas the predominant economic impact was change in community income (n = 9).

**Fig. 4:**
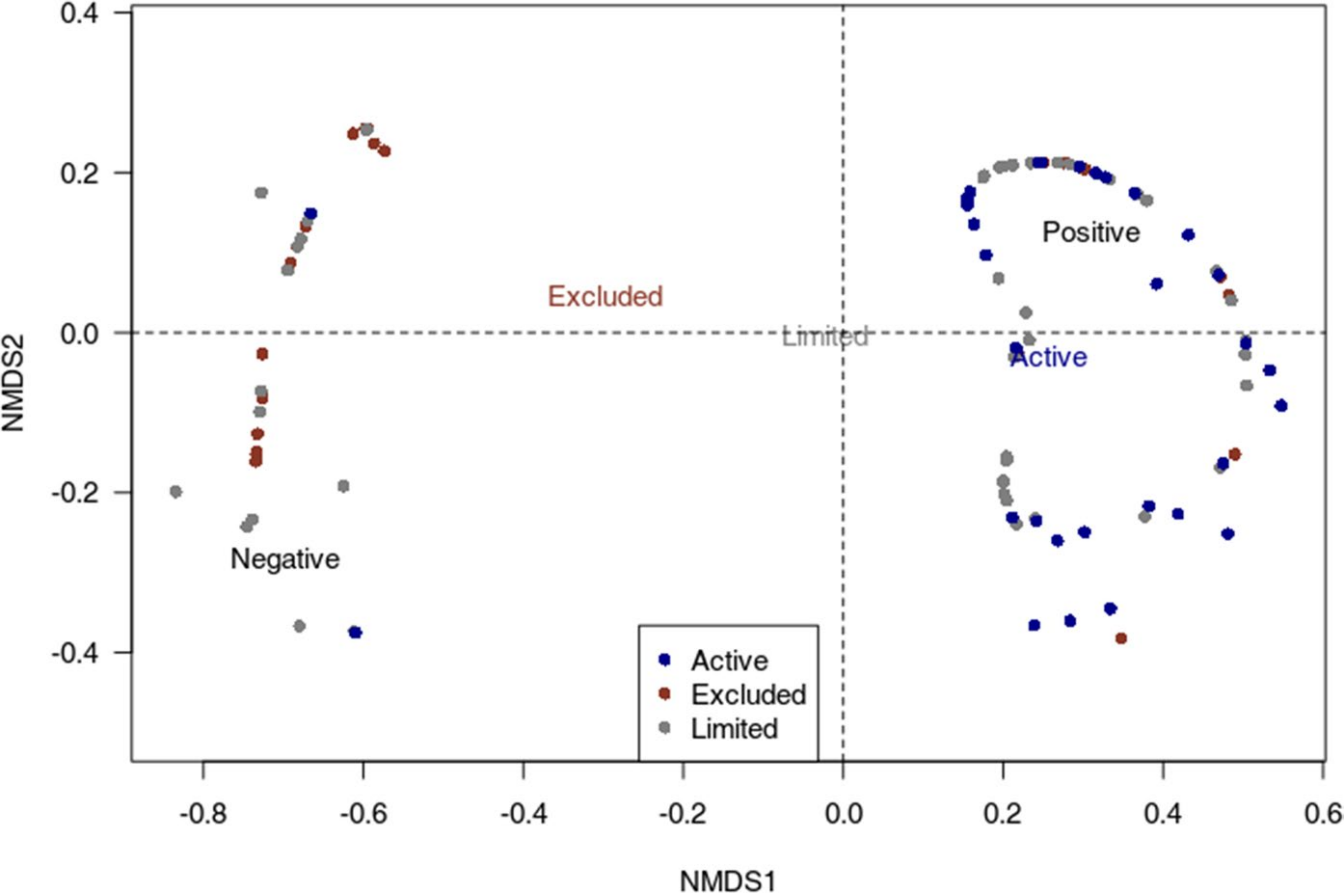
Non-metric multidimensional scaling (NMDS) ordination plot illustrating differences in impact direction among women’s level of participation in SSF management processes (i.e., excluded, limited, active). The points to the left are impacts perceived as negative, the ones to the right are positive; colours indicate women’s participation level in SSF management, and the coloured words indicate the estimated centre of each management category in this space

In this sample, 120 impacts were considered positive and 70 as negative. As an example, change in women’s leisure time was both associated to positive impacts when leisure time increased (Paul et al. 2016) and negative impacts when it decreased (Uc-Espadas et al. 2017). The NMDS analysis suggests that there is an association between women’s level of participation in SSF management and the direction of impact (Fig. 4). While women’s exclusion from SSF management was often associated to negative impacts, their active participation was mostly related to positive impacts. This is the case, for example, of Chilean women who participated in the management of a surf clam fishery in Coquimbo Bay, resulting in benefits for the whole community: “The presence of women in the present organization has helped to reduce conflicts and provide better organization” (Aburto et al. 2021, p. 5). Limited participation was associated both to negative and positive impacts. For example, Gustavsson et al. (2021) who analysed cases in Chile, France, the United Kingdom, and Tanzania, highlight how women’s marginalisation in SSF management and governance resulted in reinforcing distributive injustice. In contrast, in another study by Franco-Melendez et al. (2021) on Chilean coastal communities, women’s participation in SSF management -though limited- led to their personal empowerment. Results of a Pearson’s Chi-squared test confirmed a statistically significant relation between women’s participation level in SSF management and the report of positive impacts (*p* value = 0.001).

### Scale of the impacts of women’s participation in SSF management

**Fig. 5:**
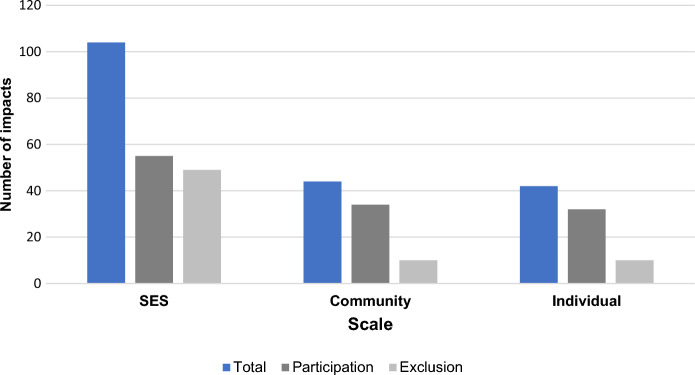
Bar chart displaying the number of reported impacts at the SES, community, and individual scales (n = 190 impacts). The impacts displayed in dark grey are associated to cases where women participate in SSF management (i.e., limited or active participation) whereas impacts in light grey are those associated to women’s exclusion cases

We found that impacts of women’s participation in SSF management affected SES, local communities, and women’s life (Fig. 5).

Most reported impacts primarily affected the whole SES (n = 104). As aforementioned, the most common one was change in the impact of management decisions on women (n = 28), followed by change in the recognition of gendered ecological knowledge (n = 20), change in the diversity of perspectives for SSF management (n = 17), change in human pressure on local ecosystems (n = 13), and change in the understanding of the gender dynamics within the SSF SES (n = 12). Because these impacts shape SSF management outcomes, they influence the dynamics of the SSF SES system. For instance, the inclusion of gendered perspectives in SSF management in Brazilian Amazonia contributed to the improved understanding of gender differences in fishing practices, thus improving SSF management outcome (Zacarkim et al. 2015).

Women’s participation in SSF management also affected local communities (n = 44), particularly through change in the social attributes of the community (n = 12), change in adaptive capacity (n = 10), and change in food security (n = 10). As an illustration, Delaney et al. (2019) show that women’s participation in a local fishery cooperative association in Japan contributed to the adaptation of their households and community to socio-economic uncertainty. Similarly, studies also documented how impacts affected women’s personal life (n = 42) through capacity building (n = 15) or women’s empowerment (n = 10), to cite the most common ones. In the latter case, we used Kabeer‘s (1999) definition of empowerment as “a process by which those who have been denied the ability to make strategic life choices acquire such an ability” (:1), interlacing the concepts of resources (both material and immaterial), agency and achievements.

## Discussion

Our work analyses the state of academic knowledge on women’s participation in SSF management and related socio-cultural, environmental, and economic impacts. Unfortunately, 40% of the studies do not report on women’s participation in SSF management. Results from the studies reporting on women’s participation in SSF management suggest that their participation is low, as most case studies report the exclusion or limited participation of women in SSF management. Our results also suggest that women’s exclusion from SSF management was associated to negative outcomes whereas women’s active participation in those processes was associated to positive outcomes. Most of the identified outputs of women’s participation were socio-cultural, bringing to light a gap on assessing the potential environmental and economic impact of women’s participation in SSF management. A final finding of this work is that most of the reported impacts unfolded at the SES scale, suggesting a win–win situation between gender-inclusive SSF management and outcomes at the SES scale.

Before commenting these results, we highlight some caveats of our work. We are aware that our review has several limitations streaming both from data availability and from the data collection methods. First, results from our work are limited by the overall lack of data on women’s contribution to the SSF sector, as also underscored in the literature (Alonso-Población & Siar 2018; Kleiber, et al. 2015; Williams 2010). We believe that the scarcity of gender information is not neutral but value-laden, since our dataset reflects authors’ interests and priorities who may chose or not to report on women’s participation in SSF management. This unavoidable authors’ subjectivity might also shape the type of reported impacts. Therefore, our results shall be interpretated with caution as they likely underestimate the total number of existing impacts associated with women’s participation in SSF management. Such data gaps represent a severe obstacle to the development of a thorough gender analysis in the fisheries sector, and we hope that our study may encourage researchers to pay more attention to gender issues and related impacts in the future. Besides, the lack of data on women’s participation in SSF management was not homogeneous across regions, but larger in Africa and in Asia, which suggests the need to strengthen research in these two regions. This is especially critical given that most of the reviewed cases were precisely located in Africa and Asia, regions where SSF is very important. Second, our results are also limited by our data collection methods, as we restricted our search to two search engines which mostly feature publications in English and exclude the wealth of studies and reports produced by non-governmental and other organizations (Kleiber et al. 2015). Such drawbacks underline the difficulty of developing comprehensive gender assessments in fisheries and reinforce the need for improving the collection of gender-disaggregated data.

### Women’s participation in SSF management

According to the reviewed literature, women’s participation in SSF management is low, with women having no or limited participation in more than 80% of cases reporting women’s participation. The finding is consistent with the work of Rabbitt et al. (2022) that also builds on Agarwal’s (2001) participation typology in the context of community-based fisheries management in Melanesia. Their results suggest that the equal number of women and men in fisheries committees, although necessary, is not sufficient to ensure the meaningful participation of women in community-based fisheries management. In the same vein, Lawless et al. (2022) found that most local organisations in the Pacific Islands region aimed to increase the number of women in traditionally male-dominated arenas without addressing structural gender inequalities. Because this approach does not displace existing gender barriers, it limits the active participation of women in SSF management and policy. Other authors have also criticized this quantitative view of women’s participation in natural resource management as a form of box-ticking approach with no transformative value (Cornwall 2003). Our results point to the fact that fisheries management is very androcentric, as also documented by other studies in SSF (Kleiber et al. 2015; Williams 2010), and echoing studies in other activity sectors such as forestry (Mwangi et al. 2011), agriculture (Buchy & Basaznew 2005; Huyer 2016)**,** wildlife conservation (Massey et al. 2022), and more generally in environmental governance systems (Alonso-Población & Siar 2018; OECD 2021). Literature on the topic identifies common barriers to women’s participation in SSF management processes such as gender norms and stereotypes and domestic responsibilities (Bradford & Katiro 2019; Galappaththi et al. 2022; Murunga 2021; SPC 2018). Our findings on women’s restricted access to SSF management and decision-making processes also echo Agarwal’s work (2001) on forestry showing that even when women can formally access community forestry groups, social norms and perceptions hinder their actual participation in meetings. In the case of patriarchal societies, women’s ability to fully participate in fisheries management is constrained owing to social norms that contribute to maintain their marginalised status and perpetrate gender inequality (Bennett 2005; Murunga 2021). To that extent, our study adds to existing evidence suggesting that many SSF communities in the world are characterized by patriarchal social structures (Bradford & Katiro 2019; Lentisco & Lee 2015). These findings imply that moving towards gender equity in SSF management will require to address structural social constructions (McDougall et al. 2021).

### Typology of impacts related to women’s participation in SSF management

One key finding of our work is the great diversity of reported socio-cultural, environmental, and economic impacts associated to women’s participation-or lack of- in SSF management. These results reinforce the need to integrate a gender perspective into SSF management and governance by looking at women’s specific engagement in those processes, as stressed by previous studies (de la Torre-Castro 2019; Galappaththi et al. 2022; Lentisco & Lee 2015).

Interestingly, we found that the most frequently reported impact- namely change in the impact of management decisions on women – was associated to the exclusion of women from SSF management and not to their participation. This finding concurs with the overall low level of women’s participation in SSF management assessed on the reviewed literature. Moreover, this result highlights how women’s exclusion from SSF management may lead to negative social consequences derived from exclusionary management, further reinforcing gender inequalities. In this sense, our findings dovetail with those from an emerging literature documenting the diverse negative impacts of gender blind SSF management policies in relation to the establishment of marine protected areas (Walker & Robinson 2009), fisheries commercialization (Hapke 2004), or access to fisheries resources (Harper et al. 2013; Siar 2003).

Another important reported impact was change in the recognition of gendered ecological knowledge through a utilitarian perspective, suggesting that researchers are more inclined to report this impact for instrumental purpose**.** This result is in line with Harper et al.’s (2013) review of women’s roles in SSF. Examining various cases in different world regions, the authors highlight the untapped value of women’s marine ecological knowledge as a source of information for fisheries management in data poor countries. Likewise, House et al.’s (2023) review of gender and participatory monitoring in community-based fisheries management (CBFM) also highlights the value of women’s knowledge for fisheries management by showing that it represents one of the main instrumental motivations for researchers on CBFM to study gender in relation to participatory monitoring. The instrumental importance of women’s ecological knowledge is not unique to SSF, but rather resonates with other research fields. In the forestry sector for instance, Agarwal (2009) shows how women’s specific knowledge on forest plant species and their harvesting methods were useful for conservation outcomes in Indian and Nepalese local forests.

Contrasting with the variety of socio-economic impacts of including women in SSF management, we recorded fewer environmental impacts. While one explanation for this finding could be that women’s participation in SSF management does not have noticeable environmental impacts, we suggest instead that women’s participation in SSF management is mostly studied through a social lens, thus challenging a thorough gender analysis of the whole SES. Such findings align with Kleiber et al. (2015), who showed that gender and fisheries research is mostly characterized by social and qualitative approaches and identified a data gap with regards to the environmental dimension of women’s fisheries-related activities. Such a gap might also reflect the common idea that women’s practices represent a low pressure on coastal ecosystems. Yet, existing evidence is too scarce to assess the actual impact of women’s fishing activities on coastal species and habitats. Indeed, the scanned existing evidence goes in both directions: in some cases, women’s extractive activities in coastal areas may be deleterious for local ecosystems (Gammage 2004), while evidence from the past suggest that in other cases certain fishing practices used by women such as clam gardens can enhance ecological outcomes in SES (Deur et al. 2015; Thrush 2006). Overall, this ecological understanding appears necessary for comprehensively assessing the health status and dynamics of marine and coastal ecosystems (Kleiber et al. 2015). Further research is thus needed for analysing women’s participation in management in relation to the sustainable use of fisheries resources.

Overall, our results suggest that women’s lack of participation in SSF management was associated to negative outcomes. These findings echo the literature in other fields, such as the work of Buchy and Basaznew (2005), showing that the absence of gender considerations in agricultural policy in Ethiopia resulted in reinforcing women’s economic marginalisation. On the contrary, women’s active participation in SSF management activities led to positive outcomes. As highlighted by some scholars, women often have recourse to informal social networks to counterbalance their restricted access to formal institutions (Agrawal 2000; Molyneux 2002; More 1990). This level of interdependency has been suggested by Westermann et al. (2005) as a driver for fostering collaboration, solidarity, and conflict resolution among women’s groups, thus bringing positive outcomes in natural resource management. Other studies have also documented positive outcomes in the SSF sector when gender norms and stereotypes are overcome and the enabling conditions are met for women to effectively participate in resource management and decision-making (de la Torre-Castro 2019; Galappaththi et al. 2022; Lentisco & Lee 2015). This suggests that encouraging women’s meaningful participation in SSF management is needed for driving positive social outcomes.

### Scale of the impacts of women’s participation in SSF management

A striking result of our study is the multiscale nature of the reported impacts, ranging from the whole SES to the individual level. Importantly, we found that a great proportion of the reported impacts unfolded at the SES scale with potential for win–win situations in SSF management. This finding aligns with previous reviews in the SSF literature suggesting that women’s participation in SSF management has knock on effects in the governance system of the SES (Galappaththi et al. 2022; Lentisco & Lee 2015). This is also consistent with studies from other resource management systems supporting that women’s participation in resource management leads to better outcomes for the whole SES and for local communities (Agarwal 2009; Westermann et al. 2005). In particular, a body of studies (Boserup 1970; Duflo 2012; Verschuur et al. 2021) and institutional reports (UN Women 2019) have documented a ripple effect from improved women’s income and economic development at the local level. Finally, our results also suggest that women’s participation in SSF management might also lead to positive change in women’s own lives. Such findings fall into the existing body of work on environmental governance and development showing how women’s participation in decision-making processes can foster their personal empowerment (de la Torre-Castro 2019) or networking capacities (Arora-Jonsson 2014). Overall, the literature identifies major enablers for women’s participation in SSF management such as state institutions (Alonso-Población & Siar 2018) and initiatives favouring women’s capacity building and self-organisation (Lentisco & Lee 2015; Murunga 2021).

Altogether, our results suggest that women’s participation in SSF management is not only necessary from an intrinsic viewpoint, but also in instrumental terms since it has the potential to contribute to improving SSF management strategies at the SES scale, while providing benefits to local communities. These results imply the need to overcome gender barriers “to ensure that women are given both a clear voice and decision-making power” (Westerman et al. 2005, p. 13). In this sense, our findings support the recent call made by scholars for a blue justice that integrates gender in SSF governance (Engen et al. 2021; TBTI 2018). The concept of blue justice proposes a shift from an economic-oriented perspective in ocean governance, the blue economy, to the recognition of social justice within those debates (Benett et al. 2021). Our article builds on this work and highlights the need for a new coastal management and ocean governance model that fully includes women in decision-making processes.

### The way forward

Given the highly gendered nature of SSF (Gallois & Duda 2016; Koralagama et al. 2017), there is a need to consider gender dimensions in its management (Harper et al. 2013; Kleiber et al. 2015). In this article, we assessed women’s participation in SSF management and related socio-cultural, environmental, and economic impacts based on existing peer-reviewed academic literature. Taken together, our findings support a better integration of gender perspectives into (1) data collection methods in fisheries research (2) SSF management and decision-making, and (3) ecological research on SSF SES.

First, we need to address the paucity of gender data in fisheries research to improve our understanding of SSF and design thorough management. One major limitation we faced in this review was the lack of gender-disaggregated data which constrained our assessment of women’s participation in SSF management, especially in regions where SSF plays a significant role for food security such as Africa and Asia (FAO 2020; Mills et al. 2011). Broadly speaking, gender myopia in SSF might result in a general underestimation of the number of fishers, fishing effort, fishing management activities, and therefore in a biased understanding of ecosystem health (Kleiber et al. 2013; Williams 2010). Second, we found that women’s participation in SSF management was low, yet their active engagement produced multiple positive outcomes. We argue that women’s active participation in SSF management and decision-making processes appears desirable for achieving gender equity in SSF and improving SSF management. Our findings align with the recent call for inclusive management in SSF, which encourages the participation of actors with diverse identities in management processes (de la Torre-Castro 2019; Nessa et al. 2020; Resurreccion 2006). For instance, women’s participation in monitoring has been recommended as a way to improve data collection, while facilitating women’s access to decision-making processes (House et al. 2023). Finally, our results identified a knowledge gap on the environmental aspects related to women’s participation in SSF management. This reflects that gender perspectives in fisheries research remain concerned by social issues and are poorly mainstreamed into environmental studies on fisheries (Kleiber et al. 2015). It is essential to foster gender knowledge in environmental research on SSF to provide a comprehensive and meaningful analysis of the role of women in SSF management.

### Supplementary Information

Below is the link to the electronic supplementary material.

Supplementary file1 (PDF 289 kb)

Supplementary file2 (PDF 222 kb)

## Data Availability

The data that support the findings of this study will shortly be openly available in Figshare repository. The data are now available oneline: Chambon, Mouna; Minarro, Sara; Alvarez-Fernandez, Santiago; Porcher, Vincent; Reyes-García, Victoria; Tonalli Drouet, huran; et al. (2023). Data for the Synthesis of Women’s Participation in Small-Scale Fisheries Management: Why Women’s Voices Matter. figshare. Dataset. 10.6084/m9.figshare.24213234v1.
