# Supplementary material for: A synthesis of women’s participation in small-scale fisheries management: why women’s voices matter
Source: Rev Fish Biol Fish. 2023 Oct 18;34(1):43–63. doi: 10.1007/s11160-023-09806-2 (PMC10838824; doi:10.1007/s11160-023-09806-2)
Supplement: Supplementary file 1 — Supplementary file1 (PDF 289 kb) [file 11160_2023_9806_MOESM1_ESM.pdf]

SUPPLEMENTARY FILE 1

Article title: A Synthesis of Women's Participation in Small-Scale Fisheries Management: Why Women's Voices Matter

Journal name: *Reviews in Fish Biology and Fisheries*

Authors and Affiliations: Mouna CHAMBON[1], Sara MIÑARRO[1], Santiago ALVAREZ FERNANDEZ[1], Vincent PORCHER[1,2], Victoria REYES-GARCIA[1,3,4], Huran TONALLI DROUET[5], Patrizia ZIVERI[1,3,6]

[1] Institute of Environmental Science and Technology, Universitat Autònoma de Barcelona, (ICTA-UAB), 08193, Bellaterra, Barcelona, Spain.

[2] Unité mixte de recherche "Savoirs Environnement Sociétés"(SENS), Institut de recherche pour le développement (IRD) & Centre de coopération internationale en recherche agronomique pour le développement (Cirad), Montpellier, France.

[3] Institució Catalana de Recerca i Estudis Avançats (ICREA), Barcelona 08010, Spain.

[4] Dept. d'Antropologia Social i Cultural, Universitat Autònoma de Barcelona, 08193, Bellaterra, Barcelona, Spain.

[5] Université libre de Bruxelles, Avenue Franklin Roosevelt 50, 1050, Bruxelles, Belgium.

[6] Dept. de Biologia Animal, Biologia Vegetal i Ecologia, Universitat Autònoma de Barcelona, 08193, Bellaterra, Barcelona, Spain.

Corresponding author:
Mouna CHAMBON
Institute of Environmental Science and Technology, Universitat Autònoma de Barcelona (ICTA-UAB), 08193, Bellaterra, Barcelona, Spain
ORCID: 0000-0002-2260-5145; Tel : (+34) 6.67.58.05.87; E-mail :Mouna.Chambon@uab.cat

Online Resource 1- Tab. S1 List of publications included in the systematic review

ID N°	Reference
**1**	Short, R.E., Mussa, J., Hill, N.A.O., Rowcliffe, M., & Milner-Gulland, E.J. (2020). Challenging assumptions: the gendered nature of mosquito net fishing and the implications for management. *Gender, Technology and Development,* 24, 66–88. DOI: 10.1080/09718524.2020.1729583
**2**	Silva, A.B., Barros, R.F., Souto, W.M., Soares, R.R., Alencar, N.L., & Lopes, C.G. (2019). "Which Fishes Do I Catch?" Predicting the Artisanal Fishers' Local Knowledge About Target-Species in Brazil. *Human Ecology, 47,* 865-876.DOI: 10.1007/s10745-019-00117-4
**3**	Barclay, K.M., Fabinyi, M., Kinch, J.P., & Foale, S.J. (2019). Governability of High-Value Fisheries in Low-Income Contexts: a Case Study of the Sea Cucumber Fishery in Papua New Guinea. *Human Ecology*. DOI: 10.1007/s10745-019-00078-8
**4**	Solano, N., Lopez-Ercilla, I., Fernandez-Rivera Melo, F.J., & Torre, J. (2021) Unveiling Women's Roles and Inclusion in Mexican Small-Scale Fisheries (SSF). *Frontiers in Marine Science,* 7, 617965. DOI: 10.3389/fmars.2020.617965
**5**	Mangubhai, S., & Lawless, S. (2021). Exploring gender inclusion in small-scale fisheries management and development in Melanesia. *Marine Policy, 123*, 104287. DOI: 10.1016/j.marpol.2020.104287
**6**	Grantham, R., Lau, J. & Kleiber, D. (2020). Gleaning: beyond the subsistence narrative. *Maritime Studies,* 19, 509–524. DOI: 10.1007/s40152-020-00200-3
**7**	Freitas, C.T., Espírito-Santo, H.M., Campos-Silva, J.V., Peres, C.A., & Lopes, P.F. (2020). Resource co-management as a step towards gender equity in fisheries. *Ecological Economics, 176*, 106709. DOI: 10.1016/j.ecolecon.2020.106709
**8**	Herrera-Racionero, P., Lizcano, E., Miret-Pastor, L., & Mascarell, Y. (2020). 'The Sea is Our Life'. Woman in the Fishery Sector of the Valencian Community. *Sociologia Ruralis*. DOI: 10.1111/soru.12318
**9**	Alati, V.M., Olunga, J., Olendo, M., Daudi, L.N., Osuka, K., Odoli, C., Tuda, P., & Nordlund, L.M. (2020). Mollusc shell fisheries in coastal Kenya: Local ecological knowledge reveals overfishing. *Ocean & Coastal Management,* 195, 105285. DOI:10.1016/j.ocecoaman.2020.105285
**10**	Purcell, S.W., Tagliafico, A., Cullis, B.R. & Gogel, B.J. (2020) Understanding Gender and Factors Affecting Fishing in an Artisanal Shellfish Fishery. *Frontiers in Marine Science,* 7, 297. DOI: 10.3389/fmars.2020.00297
**11**	Tilley, A., Burgos, A., Duarte, A. *et al.* (2021). Contribution of women's fisheries substantial, but overlooked, in Timor-Leste. *Ambio,* 50, 113–124. DOI: 10.1007/s13280-020-01335-7
**12**	Cele,N. (2020) Are you a fisher or mussel collector?: Examining gendered identity markers in the small-scale fishing industry. *Agenda,* 34, 1, 141-150. DOI: 10.1080/10130950.2020.1721195
**13**	da Silva Mourão, J., Baracho, R.L., Martel, G. *et al.* (2020). Local ecological knowledge of shellfish collectors in an extractivist reserve, Northeast Brazil: implications for co-management. *Hydrobiologia,* 847, 1977–1997. DOI: 10.1007/s10750-020-04226-w
**14**	Delaney, A.E., Schreiber, M.A. & Alfaro-Shigueto, J. (2019). Innovative and traditional actions. *Maritime Studies,* 18, 287–295. DOI: 10.1007/s40152-019-00150-5
**15**	Samoilys, M.A., Osuka, K.E., Mussa, J., Rosendo, S., Riddell, M., Diade, M., Mbugua, J., Kawaka, J.A., Hill, N., & Koldewey, H.J. (2019). An integrated assessment of coastal fisheries in Mozambique for conservation planning. *Ocean & Coastal Management, 182*, 104924. DOI: 10.1016/j.ocecoaman.2019.104924
**16**	Manyungwa, C.L., Hara, M.M. & Chimatiro, S.K. (2019). Women's engagement in and outcomes from small-scale fisheries value chains in Malawi: effects of social relations. *Maritime Studies*, 18, 275–285. DOI: 10.1007/s40152-019-00156-z
**17**	Singleton, R.L., Allison, E.H., Gough, C., Kamat, V.R., leBillon, P., Robson, L.C., & Sumaila, U.R. (2019). Conservation, contraception and controversy: Supporting human rights to enable sustainable fisheries in Madagascar. *Global Environmental Change*. DOI: doi.org/10.1016/j.gloenvcha.2019.101946
**18**	Siegelman, B., Haenn, N.H., & Basurto, X. (2019). "Lies build trust": Social capital, masculinity, and community-based resource management in a Mexican fishery. *World Development,* 123, 104601, ISSN 0305-750X.DOI: 10.1016/j.worlddev.2019.05.031
**19**	Kwok, Y.E., KC, K.B., Silver, J.J. and Fraser, E. (2020), Perceptions of gender dynamics in small-scale fisheries and conservation areas in the Pursat province of Tonle Sap Lake, Cambodia. *Asia-Pacific View*, 61, 54-70. DOI : 10.1111/apv.12225
**20**	Furkon, Nessa, N., Ambo-Rappe, R. *et al.* (2020). Social-ecological drivers and dynamics of seagrass gleaning fisheries. *Ambio,* 49, 1271–1281. DOI: 10.1007/s13280-019-01267-x
**21**	Rabbitt, S., Lilley, I., Albert, S., & Tibbetts, I.R. (2019). What's the catch in who fishes? Fisherwomen's contributions to fisheries and food security in Marovo Lagoon, Solomon Islands. *Marine Policy*, 108, 103667. DOI:10.1016/j.marpol.2019.103667
**22**	Wosu, A. (2019). Access and institutions in a small-scale octopus fishery: A gendered perspective. *Marine Policy,* 108, 103649. DOI : 10.1016/j.marpol.2019.103649

**23**	Millar, J., Robinson, W.A., Baumgartner, L.J., Homsombath, K., Chittavong, M., Phommavong, T., & Singhanouvong, D. (2018). Local perceptions of changes in the use and management of floodplain fisheries commons: the case of Pak Peung wetland in Lao PDR. *Environment, Development and Sustainability*, 1-18, 1835–1852. DOI: 10.1007/s10668-018-0105-3
**24**	Lavoie, A., Lee, J., Sparks, K., Hoseth, G., & Wise, S. (2019). Engaging with Women's Knowledge in Bristol Bay Fisheries through Oral History and Participatory Ethnography. *Fisheries*, 44, 331-337. DOI: 10.1002/fsh.10271
**25**	Gianelli, I., Ortega, L., Defeo, O. (2019). Modeling short-term fishing dynamics in a small-scale intertidal shellfishery. *Fisheries Research*, 209, 242–250. DOI: 10.1016/j.fishres.2018.09.028
**26**	Khan, F.N., Collins, A.M., Nayak, P.K. *et al.* (2018). Women's perspectives of small-scale fisheries and environmental change in Chilika lagoon, India. *Maritime Studies,* 17, 145–154. DOI: 10.1007/s40152-018-0100-1
**27**	Rohe, J., Schlüter, A., & Ferse, S.C.A. (2018). A gender lens on women's harvesting activities and interactions with local marine governance in a South Pacific fishing community. *Maritime Studies,* 17, 155–162. DOI: 10.1007/s40152-018-0106-8
**28**	Kleiber, D., Harris, L., & Vincent, A.C.J. (2018). Gender and marine protected areas: a case study of Danajon Bank, Philippines. *Maritime Studies,* 17, 163–175. DOI: 10.1007/s40152-018-0107-7
**29**	Gallardo-Fernández, G.L., & Saunders, F. (2018). "Before we asked for permission, now we only give notice": Women's entrance into artisanal fisheries in Chile. *Maritime Studies,* 17, 177–188. DOI: 10.1007/s40152-018-0110-z
**30**	Purcell, S.W., Lalavanua, W., Cullis, B.R., & Cocks, N.A. (2018). Small-scale fishing income and fuel consumption: Fiji's artisanal sea cucumber fishery. *ICES Journal of Marine Science, 75*, 1758–1767. DOI: 10.1093/icesjms/fsy036
**31**	Quiros, T.E., Beck, M.W., Araw, A., Croll, D.A., & Tershy, B.R. (2018). Small-scale seagrass fisheries can reduce social vulnerability: a comparative case study. *Ocean & Coastal Management*, 157, 56-67. DOI :10.1016/j.ocecoaman.2018.02.003
**32**	Uc-Espadas, M., Molina-Rosales, D., Gurri, F.D., Pérez-Jiménez, J.C., & Vázquez-García, V. (2018). Fishing activities by gender and reproductive stage in Isla Arena, Campeche, Mexico. *Marine Policy,* 89, 34–39. DOI: 10.1016/j.marpol.2017.12.011
**33**	Drury O'Neill, E., Crona, B., Ferrer, A.J.G., Pomeroy, R., & Jiddawi, N.S. (2018). Who benefits from seafood trade? A comparison of social and market structures in small-scale fisheries. *Ecology and Society*, 23. DOI:10.5751/es-10331-230312
**34**	Rivera, V.S., Cordero, P.M., Rojas, D.C. *et al. (2017).* Institutions and collective action in a Costa Rican small-scale fishery cooperative: the case of CoopeTárcoles R.L. *Maritime Studies,* 16, 22. DOI: 10.1186/s40152-017-0077-1
**35**	Fadigas, A.B. (2017). Vulnerability factors of shellfisherwomen in the face of oil spill events: An analysis of the Prestige case. *International journal of disaster risk reduction, 24,* 560-567. DOI: 10.1016/j.ijdrr.2017.07.010
**36**	Locke, C., Muljono, P., Mcdougall, C., & Morgan, M. (2017). Innovation and gendered negotiations: Insights from six small-scale fishing communities. *Fish and Fisheries,* 18, 943–957. DOI: 10.1111/faf.12216
**37**	Uc-Espadas, M., Molina-Rosales, D., Vázquez-García, V., Pérez-Jiménez, J. C., & Gurri-García, F. (2017). Fishing permits and gender relations in Isla Arena, Campeche. *Agricultura, sociedad y desarrollo, 14*(3), 383-404. Recuperado en 15 de septiembre de 2022, de http://www.scielo.org.mx/scielo.php?script=sci_arttext&pid=S1870-54722017000300383&lng=es&tlng=en.
**38**	Drury O'Neill, D, D.E., & Crona, B. (2017). Assistance networks in seafood trade – A means to assess benefit distribution in small-scale fisheries. *Marine Policy,* 78, 196-205. DOI: 10.1016/j.marpol.2017.01.025.
**39**	Purdy, D.H., Hadley, D.J., J Kenter, J.O, & Kinch, J.(2017). Sea Cucumber Moratorium and Livelihood Diversity in Papua New Guinea. *Coastal Management,* 45,2, 161-177. DOI: 10.1080/08920753.2017.1278147
**40**	McClanahan, T.R., & Abunge, C. (2017). Fish trader's gender and niches in a declining coral reef fishery: implications for sustainability, *Ecosystem Health and Sustainability,* 3,6. DOI: 10.1080/20964129.2017.1353288
**41**	Paul, S.A.L., Wilson, A.M.W., Cachimo, R., & Riddell, M.A. (2016). Piloting participatory smartphone mapping of intertidal fishing grounds and resources in northern Mozambique: Opportunities and future directions. *Ocean & Coastal Management,* 134, 79–92. DOI: 10.1016/j.ocecoaman.2016.09.018
**42**	Purcell, S.W., Ngaluafe, P., Aram, K.T., & Lalavanua, W. (2016). Trends in small-scale artisanal fishing of sea cucumbers in Oceania. *Fisheries Research, 183,* 99-110. DOI: 10.1016/j.fishres.2016.05.010
**43**	Andrade, L.P., Silva-Andrade, H.M.L., Lyra-Neves, R.M. *et al.* (2016). Do artisanal fishers perceive declining migratory shorebird populations? *Journal of Ethnobiology and Ethnomedicine, 12,* 16. DOI: 10.1186/s13002-016-0087-x
**44**	Gianelli, I., Martínez, G., & Defeo, O. (2015). An ecosystem approach to small-scale co-managed fisheries: The yellow clam fishery in Uruguay. *Marine Policy*, 62. 196-202. DOI: 10.1016/j.marpol.2015.09.025.
**45**	Santos, A., N. (2015). Fisheries as a way of life: Gendered livelihoods, identities and perspectives of artisanal fisheries in eastern Brazil. *Marine Policy*, Elsevier, 62(C), 279-288. DOI: 10.1016/j.marpol.2015.09.007
**46**	Nunan, F., Hara, M., Onyango, P. (2015). Institutions and Co-Management in East African Inland and Malawi Fisheries: A Critical Perspective. *World Development,* 70, 203–214. DOI: 10.1016/j.worlddev.2015.01.009
**47**	Zacarkim, C.E., Piana, P.A., Baumgartner, G. *et al.* (2015). The panorama of artisanal fisheries of the Araguaia River, Brazil. *Fisheries Science*, 81, 409–416. DOI: 10.1007/s12562-015-0853-z

**48**	Ramírez, A., Ortiz, M., Steenbeek, J., & Christensen, V. (2015). Evaluation of the effects on rockfish and kelp artisanal fisheries of the proposed Mejillones Peninsula marine protected area (northern Chile, SE Pacific coast). *Ecological Modelling*, 297, 141–153. DOI: 10.1016/j.ecolmodel.2014.11.012
**49**	Thorpe, A., Pouw, N., Baio, A., Sandi, R., Ndomahina, E.T., & Lebbie, T. (2014). "Fishing Na Everybody Business": Women's Work and Gender Relations in Sierra Leone's Fisheries. *Feminist Economics*, 20, 53–77. DOI:10.1080/13545701.2014.895403
**50**	Kleiber, D., Harris, L. M., & Vincent, A.C.J. (2013). Improving fisheries estimates by including women's catch in the Central Philippines. *Canadian Journal of Fisheries and Aquatic Sciences*, 71, 5, 656-664. DOI: 10.1139/cjfas-2013-0177
**51**	Matsue, N., Daw, T., Garrett, L. (2014). Women Fish Traders on the Kenyan Coast: Livelihoods, Bargaining Power, and Participation in Management. *Coastal Management*, 42, 531–554. DOI: 10.1080/08920753.2014.964819
**52**	Fröcklin, S., De La Torre-Castro, M., Lindström, L., & Jiddawi, N.S. (2013). Fish Traders as Key Actors in Fisheries: Gender and Adaptive Management. *Ambio*, 42, 951–962. DOI :10.1007/s13280-013-0451-1
**53**	Harper, S., Zeller, D., Hauzer, M., Pauly, D., & Sumaila, U.R. (2013). Women and fisheries: Contribution to food security and local economies. *Marine Policy*, 39, 56–63. DOI: 10.1016/j.marpol.2012.10.018
**54**	Hauzer, M., Dearden, P., & Murray, G. (2013). The fisherwomen of Ngazidja island, Comoros: Fisheries livelihoods, impacts, and implications for management. *Fisheries Research*, 140, 28–35. DOI: 10.1016/j.fishres.2012.12.001
**55**	Neis, B., Gerrard, S., & Power, N.G. (2013). Women and Children First: the Gendered and Generational Social-ecology of Smaller-scale Fisheries in Newfoundland and Labrador and Northern Norway. *Ecology and Society*, 18. DOI: 10.5751/es-06010-180464
**56**	Ngwenya, B.N., Mosepele, K.K., & Magole, L. (2012). A case for gender equity in governance of the Okavango Delta fisheries in Botswana. *Natural Resources Forum*, 36, 109–122. DOI:10.1111/j.1477-8947.2012.001450.x
**57**	Crawford, B., Herrera, M.D., Hernandez, N., Leclair, C.R., Jiddawi, N., Masumbuko, S., Haws, M. (2010). Small Scale Fisheries Management: Lessons from Cockle Harvesters in Nicaragua and Tanzania. *Coastal Management*, 38, 195–215. DOI : 10.1080/08920753.2010.483174
**58**	Arce-Ibarra, A.M., Charles, A.T. (2008). Inland fisheries of the Mayan Zone in Quintana Roo, Mexico: Using a combined approach to fishery assessment for data-sparse fisheries. *Fisheries Research*, 91, 151–159. DOI: 10.1016/j.fishres.2007.11.015
**59**	Arif, M., Subramaniam, T., & Raduan, M.S. (2008). Bajau women as key workforce in artisanal fishermen family in mengkabong village, tuaran, sabah.
**60**	Di Ciommo, R. C. (2007). Gender, Tourism, and Participatory Appraisals at the Corumbau Marine Extractive Reserve, Brazil. *Human Ecology Review*, *14*, 1, 56–67. http://www.jstor.org/stable/24707643
**61**	Kuster, C., Vuki, V.C., & Zann, L.P. (2005). Long-term trends in subsistence fishing patterns and coral reef fisheries yield from a remote Fijian island. *Fisheries Research*, 76, 221–228. DOI: 10.1016/j.fishres.2005.06.011
**62**	Ashworth, J.S., Ormond, R.F.G., & Sturrock, H.T. (2004). Effects of reef-top gathering and fishing on invertebrate abundance across take and no-take zones. *Journal of Experimental Marine Biology and Ecology*, 303, 221–242. DOI: 10.1016/j.jembe.2003.11.017
**63**	Branch, G.M., May, J., Roberts, B., Russell, E., & Clark, B.M. (2002). Case studies on the socio-economic characteristics and lifestyles of subsistence and informal fishers in South Africa. *South African Journal of Marine Science*, 24, 439–462. DOI: 10.2989/025776102784528457
**64**	King, M. & Faasili U. (1999). Community-based management of subsistence fisheries in Samoa: Fisheries Management and Ecology, 6, 2, 133-144. DOI: doi.org/10.1046/j.1365-2400.1999.00136.x
**65**	Uduji, J.I., & Okolo-Obasi, E.N. (2020). Does corporate social responsibility (CSR) impact on development of women in small-scale fisheries of sub-Saharan Africa? Evidence from coastal communities of Niger Delta in Nigeria. *Marine Policy*, 118. DOI:10.1016/j.marpol.2018.10.036
**66**	María Esther, C.-C., Miguel Ángel, C.-M., Gabriela, M.-M., Ileana, E., Andrés Miguel, C.-M., Luis, M.-C. (2021). Analysis of the Gulf of California cannonball jellyfish fishery as a complex system. *Ocean & Coastal Management* 207, 105610. DOI: 10.1016/j.ocecoaman.2021.105610
**67**	Barrero-Amórtegui, Y., & Maldonado, J.H. (2021). Gender composition of management groups in a conservation agreement framework: Experimental evidence for mangrove use in the Colombian Pacific. *World Development* 142, 105449. DOI: 10.1016/j.worlddev.2021.105449
**68**	Thomas, A., Mangubhai, S., Fox, M., Meo, S., Miller, K., Naisilisili, W., Veitayaki, J., & Waqairatu, S. (2021). Why they must be counted: Significant contributions of Fijian women fishers to food security and livelihoods. *Ocean & Coastal Management*, 205, 105571. DOI: 10.1016/j.ocecoaman.2021.105571
**69**	Kaminski, A.M., Cole, S.M., Al Haddad, R.E., Kefi, A.S., Chilala, A.D., Chisule, G., Mukuka, K.N., Longley, C., Teoh, S.J., Ward, A.R. (2020). Fish Losses for Whom? A Gendered Assessment of Post-Harvest Losses in the Barotse Floodplain Fishery, Zambia. *Sustainability*, 12, 10091. DOI: 10.3390/su122310091

**70**	Nessa, N., Gatta, R., Ambo-Rappe, R., Jompa, J., & Yahya, A. (2020). The role of women in the utilization of Enhalus acoroides: livelihoods, food security, impacts and implications for coastal area management. IOP Conference Series: *Earth and Environmental Science*, 564. DOI: 10.1088/1755-1315/564/1/012073
**71**	Cezar, L. S., & Theis, R. (2020). Ser ou não ser pescadora artesanal? Trabalho feminino, reconhecimento e representação social entre marisqueiras da Bacia de Campos, RJ. *Revista de Antropologia*, 63, 3, 1–20. https://www.jstor.org/stable/27008100
**72**	Torell, E., Bilecki, D., Owusu, A., Crawford, B., Beran, K., & Kent, K. (2019). Assessing the Impacts of Gender Integration in Ghana's Fisheries Sector. *Coastal Management*, 47, 507–526. DOI :10.1080/08920753.2019.1669098
**73**	MacKeracher, T., Foale, S. J., Gurney, G. G., & Purcell, S. W. (2019). Adoption and diffusion of technical capacity-building innovations by small-scale artisanal fishers in Fiji. *Ecology and Society*, 24, 2. https://www.jstor.org/stable/26796932
**74**	Musinguzi, L., Natugonza, V., Efitre, J., & Ogutu-Ohwayo R. (2018) The role of gender in improving adaptation to climate change among small-scale fishers. *Climate and Development*, 10, 6, 566-576. DOI: 10.1080/17565529.2017.1372262
**75**	Hoque Mozumder, M.M., Shamsuzzaman, M.M., Rashed-Un-Nabi, M., Karim, E. (2018). Social-ecological dynamics of the small- scale fisheries in Sundarban Mangrove Forest, Bangladesh. *Aquaculture and Fisheries,* 3, 38–49. DOI :10.1016/j.aaf.2017.12.002
**76**	Mul, M., Pettinotti, L., Amonoo, N. A., Bekoe-Obeng, E., & Obuobie, E. (2017). Dependence of riparian communities on ecosystem services in northern Ghana. Colombo, Sri Lanka: International Water Management Institute (IWMI). 43p. (IWMI Working Paper 179). DOI: 10.5337/2018.201
**77**	Lowe, M.E. (2015). Localized practices and globalized futures: challenges for Alaska coastal community youth. *Maritime Studies*, 14. DOI:10.1186/s40152-015-0024-y
**78**	Eder, J.F. (2012). The role of gender in the reduction of fishing effort in the coastal Philippines. *Gender and Sustainability: Lessons from Asia and Latin America*. 187-206.
**79**	Gurung, T.B. (2007). Restoration of small lakes through cooperative management: A suitable strategy for poverty-laden areas in developing countries? *Lakes & Reservoirs: Science, Policy and Management for Sustainable Use*, 12, 237–246. DOI: 10.1111/j.1440-1770.2007.00341.x
**80**	Di Ciommo, R. (2007). Fishermen and fisherwomen: gender equity in a marine extractive reserve. *Ambiente & Sociedade*, 10, 1, 151-163. DOI: 10.1590/S1414-753X2007000100010
**81**	Ferguson, C.E. (2021) A Rising Tide Does Not Lift All Boats: Intersectional Analysis Reveals Inequitable Impacts of the Seafood Trade in Fishing Communities. Frontiers in Marine Science, 8,625389. DOI: 10.3389/fmars.2021.625389
**82**	Purcell, S. W., Tagliafico,A., Cullis, B. R., & Gogel. B.J., (2021). Socioeconomic impacts of resource diversification from small-scale fishery development. *Ecology and Society*, 26,1,14. DOI: 10.5751/ES-12183-260114
**83**	Zeller, D., Vianna, G.M.S., Ansell, M., Coulter, A., Derrick, B., Greer, K., Noël, S-L., Palomares, M.L.D., Zhu, A., & Pauly, D. (2021). Fishing Effort and Associated Catch per Unit Effort for Small-Scale Fisheries in the Mozambique Channel Region: 1950–2016. Frontiers in Marine Science, 8, 707999. DOI: 10.3389/fmars.2021.707999
**84**	Gustavsson, M., Frangoudes, K., Lindström, L., Álvarez Burgos, M.C., & de la Torre-Castro, M. (2021). Gender and Blue Justice in small-scale fisheries governance. *Marine Policy,* 133, 104743. DOI:10.1016/j.marpol.2021.104743
**85**	Pedroza-Gutiérrez, C., & Hapke, H.M. (2021). Women's work in small-scale fisheries: a framework for accounting its value. *Gender, Place & Culture,* 1–18. DOI :10.1080/0966369x.2021.1997936
**86**	Aburto, J.A., Stotz, W., Cundill, G., Tapia, C. (2021). Toward understanding the long-term persistence of a local governance system among artisanal fishers in Chile. *Ecology and Society,* 26. DOI:10.5751/es-12479-260305
**87**	Bernos T.A., Travouck C., Ramasinoro N., Fraser D.J., & Mathevon B. (2021) What can be learned from fishers' perceptions for fishery management planning? Case study insights from Sainte-Marie, Madagascar. *PLoS ONE,* DOI: 10.1371/journal.pone.0259792.
**88**	Jeeva, J. C. & Gopal, N. (2021). A Participatory Assessment of Entrepreneurial Skills among Women in Small-Scale Fisheries. *FISH Technologies.* 58,3, 171-176. DOI: 10.3389/fmars.2020.6179
**89**	Torell, E., Manyungwa-Pasani, C., Bilecki, D., Gumulira, I., Yiwombe, G. (2021). Assessing and Advancing Gender Equity in Lake Malawi's Small-Scale Fisheries Sector. *Sustainability;* 13,23,13001. DOI: 10.3390/su132313001
**90**	Franco-Meléndez, M., Tam, J., van Putten, I., Cubillos, L.A. (2021). Integrating human and ecological dimensions: The importance of stakeholders' perceptions and participation on the performance of fisheries co-management in Chile. *PLOS ONE,* 16,8, e0254727. DOI: 10.1371/journal.pone.0254727
**91**	Thomas, A., Mangubhai, S., Fox, M., Meo, S., Miller, K., Naisilisili, W., Veitayaki, J., & Waqairatu, S. (2021). Why they must be counted: Significant contributions of Fijian women fishers to food security and livelihoods. *Ocean & Coastal Management,* 205, 105571. DOI: 10.1016/j.ocecoaman.2021.105571
**92**	Da Silva Mourão, J., Baracho, R.L., De Faria Lopes, S., Medeiros, M.C., & Diele, K. (2021). The harvesting process and fisheries production of the venus clam Anomalocardia flexuosa in a Brazilian extractive reserve, with implications for gender-sensitive management. *Ocean & Coastal Management,* 213, 105878. DOI: 10.1016/j.ocecoaman.2021.105878

**93**	Lau, J.D., Sutcliffe, S.R., Barnes, M.L., Mbaru, E.K., Muly, I., Muthiga, N.A., Wanyonyi, S., & Cinner, J.E. (2021). COVID-19 impacts on coastal communities in Kenya. *Marine Policy*, 134, 104803 - 104803. DOI: 10.1016/j.marpol.2021.104803
**94**	Swathi Lekshmi, P.S., Kalidoss Radhakrishnan, R., Narayanakumar, V., Vipinkumar, P., Shinoj Parappurathu, Shyam, S., Salim, Johnson, B., & Phalguni , P. (2022).Gender and small-scale fisheries: Contribution to livelihood and local economies. *Marine Policy,* 136, 104913. DOI: 10.1016/j.marpol.2021.104913.
**95**	Silva, M.R.O., Silva, A.B., Barbosa, J.B., Cássia Amaral, P., & Lopes, F.M. (2022). Empowering fisherwomen leaders helped reduce the effects of the COVID-19 pandemic on fishing communities: Insights from Brazil. *Marine Policy*, 135, 104842, ISSN 0308-597X. DOI: 10.1016/j.marpol.2021.104842.
**96**	Yadav, S., Fisam, A., Dacks, R., Madin, J. S., & Mawyer, A. (2021). Shifting fish consumption preferences can impact coral reef resilience in the Maldives: a case study. *Marine Policy*, 134, 104773. DOI: 10.1016/j.marpol.2021.104773
**97**	Rice WS (2022). Exploring common dialectical tensions constraining collaborative communication required for post-2020 conservation. *Journal of Environmental Management,* 316, 115187. DOI: 10.1016/j.jenvman.2022.115187. Epub 2022 May 11. PMID: 35561492.
**98**	Chitará-Nhandimo, S., Chissico, A., Mubai, M.E., Cabral, A.D.S., Guissamulo, A., & Bandeira, S. (2022). Seagrass Invertebrate Fisheries, Their Value Chains and the Role of LMMAs in Sustainability of the Coastal Communities—Case of Southern Mozambique. *Diversity* 14, 170. DOI :10.3390/d14030170
**99**	Berenji, S., Nayak, P.K., & Shukla, A. (2021). Exploring Values and Beliefs in a Complex Coastal Social-Ecological System: A Case of Small-Scale Fishery and Dried Fish Production in Sagar Island, Indian Sundarbans. Frontiers in Marine Science, 8, 795973. DOI: 10.3389/fmars.2021.795973
**100**	Sreya, P.S., Parayil, C., Aswathy, N., Bonny, B.P., Aiswarya, T.P., & Nameer, P.O. (2021). Economic vulnerability of small-scale coastal households to extreme weather events in Southern India. *Marine Policy*, 131, 104608. DOI: 10.1016/j.marpol.2021.104608
**101**	López-Ercilla, I., Espinosa-Romero, M.J., Rivera-Melo, F.F., Fulton, S., Fernández, R., Torre, J., Acevedo-Rosas, A., Hernández-Velasco, A., & Amador, I. (2021). The voice of Mexican small-scale fishers in times of COVID-19: impacts, responses, and digital divide. *Marine Policy*, 131, 104606. DOI: 10.1016/j.marpol.2021.104606
**102**	Oloko, A., Fakoya, K., Ferse, S., Breckwoldt, A., & Harper, S. (2022) The Challenges and Prospects of Women Fisherfolk in Makoko, Lagos State, Nigeria. *Coastal Management*, 50, 2, 124-141. DOI: 10.1080/08920753.2022.2022969
**103**	Casagrande, A., & Rover, O.J. (2021). Institucionalização de pescarias artesanais e diálogo equitativo: o caso do molusco berbigão (Anomalocardia brasiliana) na Reserva Extrativista Marinha do Pirajubaé. *Desenvolvimento e Meio Ambiente,* 58. DOI : 10.5380/dma.v58i0.75245

Online Resource 2- Tab.S2 Justification for document's exclusion from the second screening process (n=24)

Reference	Reason for exclusion
Kluger, L.C., Alff, H., Alfaro-Córdova, E., & Alfaro-Shigueto, J. (2020). On the move: The role of mobility and migration as a coping strategy for resource users after abrupt environmental disturbance – the empirical example of the Coastal El Niño 2017. *Global Environmental Change,* 63, 102095. DOI : 10.1016/j.gloenvcha.2020.102095	Not meeting criterion *ii*
Paul, T.T., Salim, S.S., S, M., Sarkar, U.K., & Das, B.K. (2020). Understanding variations in socio-economic vulnerabilities and the strategies adopted by small scale fishing communities of tropical reservoirs. *Fisheries Research,* 226, 105523. DOI : 10.1016/j.fishres.2020.105523	Not meeting criterion *iv*
Bradford, K., & Katikiro, R.E. (2019). Fighting the tides: A review of gender and fisheries in Tanzania. *Fisheries Research*, 216, 79–88. DOI: 10.1016/j.fishres.2019.04.003	Not meeting criterion *i*
de la Torre-Castro, M. (2019). Inclusive Management Through Gender Consideration in Small-Scale Fisheries: The Why and the How. Frontiers in Marine Science, 6, 156. DOI: 10.3389/fmars.2019.00156	Not meeting criterion *i*
Fulton S., Hernández, A., Suárez-Castillo, A., Fernández-Rivera Melo, F., Rojo, M., Sáenz-Arrollo, A., Hudson Weaver, A., Cudney-Bueno, R., Micheli, F., & Torre., J. (2019). From fishing fish to fishing data: the role of artisanal fishers in conservation and resource management in Mexico. En: Viability and sustainability of small-scale fisheries in Latin America and The Caribbean. S Salas, M.J. Barragán-Paladines, R. Chuenpagdee(eds.). MARE Publication Series, 19. Springer, Cham. pp. 151-175.	Not meeting criterion *iv*
Lauria, V., Das, I., Hazra, S., Cazcarro, I., Arto, I., Kay, S., Ofori-Danson, P.K., Ahmed, M., Hossain, M.A., Barangé, M., & Fernandes, J.A. (2018). Importance of fisheries for food security across three climate change vulnerable deltas. *The Science of the total environment,* 640-641, 1566-1577. DOI : 10.1016/j.scitotenv.2018.06.011	Not meeting criterion *i*
Hurley, M., & Manel, C. (2015). Coordination, Development and Governance of Senegal Small-Scale Fisheries.	Not meeting criterion *iv*
Sharma, C.M. (2008) Freshwater Fishes, Fisheries and Habitat Prospects of Nepal. *Aquatic Ecosystem, Health and Management,* 11, 75-82. DOI: 10.1080/14634980802317329	Not meeting criterion *iv*
Stacey, N., Gibson, E., Loneragan, N.R., Warren, C., Wiryawan, B., Adhuri, D., & Fitriana, R. (2019). Enhancing coastal livelihoods in Indonesia: an evaluation of recent initiatives on gender, women and sustainable livelihoods in small-scale fisheries. *Maritime Studies,* 18, 359–371. DOI:10.1007/s40152-019-00142-5	Not meeting criterion *i*
Furtado, L.G., Leitão, W.M., & Mello, A.F. (1993). *Povos das águas : realidade e perspectivas na Amazônia.*	Not meeting criterion *i*
Purcell, S.W., Fraser, N.J., Tagica, S., Lalavanua, W., & Ceccarelli, D.M. (2018) Discriminating Catch Composition and Fishing Modes in an Artisanal Multispecies Fishery. Frontiers in Marine Science, 5, 243. DOI : 10.3389/fmars.2018.00243	Redundancy of data: based on the same data than ID 30
Purcell, S.W., Ngaluafe, P., Foale, S.J., Cocks, N., Cullis, B.R., Lalavanua, W. (2016) Multiple Factors Affect Socioeconomics and Wellbeing of Artisanal Sea Cucumber Fishers. *PLoS ONE* 11, 12, e0165633. DOI: 10.1371/journal.pone.0165633	Redundancy of data: based on the same data than ID 42
Amadu, I., Armah, F.A., & Aheto, D.W. (2021). Assessing Livelihood Resilience of Artisanal Fisherfolk to the Decline in Small-Scale Fisheries in Ghana. *Sustainability,* 13, 10404. DOI:10.3390/su131810404	Not meeting criteria *iii* and *iv*
Amadu, I., Armah, F.A., Aheto, D.W., Adongo, A.C. (2021). A study on livelihood resilience in the small-scale fisheries of Ghana using a structural equation modelling approach. *Ocean & Coastal Management*,215, 105952, ISSN 0964-5691. DOI: 10.1016/j.ocecoaman.2021.105952.	Not meeting criterion *iv*
Engen, S., Hausner, V.H., Gurney, G.G., Broderstad, E.G., Keller, R., Lundberg, A.K., Murguzur, F.J.A., Salminen, E., Raymond, C.M., Falk-Andersson, J., & Fauchald, P. (2021). Blue justice: A survey for eliciting perceptions of environmental justice among coastal planners' and small-scale fishers in Northern-Norway. *PLOS ONE*, 16, e0251467. DOI: 10.1371/journal.pone.0251467	Not meeting criterion *iv*
Stacey, N., Gibson, E., Loneragan, N.R., Warren, C., Wiryawan, B., Adhuri, D.S., Steenbergen, D.J., & Fitriana, R. (2021). Developing sustainable small-scale fisheries livelihoods in Indonesia: Trends, enabling and constraining factors, and future opportunities. *Marine Policy*, 132, 104654. DOI: doi.org/10.1016/j.marpol.2021.104654	Not meeting criterion *i*
Lawless, S., Cohen, P. J., McDougall, C., Mangubhai, S., Song, A. M. and Morrison, T. H. (2022). Tinker, tailor or transform: Gender equality amidst social-ecological change. *Global Environmental Change*, 72. 102434, ISSN 0959-3780. DOI: 10.1016/j.gloenvcha.2021.102434.	Redundancy of data: based on the same

	data than ID 5
Mangubhai, S., Nand, Y., Reddy, C., Jagadish, A. (2021). Politics of vulnerability: Impacts of COVID-19 and Cyclone Harold on Indo-Fijians engaged in small-scale fisheries. Environmental Science and Policy, 120:195-203. DOI: 10.1016/j.envsci.2021.03.003. Epub 2021 Mar 25. PMID: 34867082; PMCID: PMC8629164.	Not meeting criterion *iv*
Kimbu, A.N., Booyens, I., & Winchenbach, A. (2022). Livelihood Diversification Through Tourism: Identity, Well-being, and Potential in Rural Coastal Communities. *Tourism Review International*, 26, 25–40. DOI:10.3727/154427221x16245632411854	Not meeting criterion *iii*
Smallhorn-West, P., Van Der Ploeg, J., Boso, D., Sukulu, M., Leamae, J., Isihanua, M., Jasper, M., Saeni-Oeta, J., Batalofo, M., Orirana, G., Konamalefo, A., Houma, J., & Eriksson, H. (2022). Patterns of catch and trophic signatures illustrate diverse management requirements of coastal fisheries in Solomon Islands. *Ambio,* 51, 1504–1519. DOI:10.1007/s13280-021-01690-z	Not meeting criterion *iv*
Massey, L.M., McCord Camerden, P., Gaos, A.R., Liles, M.J., Seminoff, A.J., & Ahern, A.L.M (2022). Challenging gender inequity in wildlife conservation: a women's group leading sea turtle conservation efforts in El Salvador. *Local Environment*, 27,1, 1-15. DOI: 10.1080/13549839.2021.1997962	Not meeting criterion *iii*
Lee, J., & Kim, D. (2021). Analysis of the Discriminatory Perceptions of Victims on Damage from Environmental Pollution: A Case Study of the Hebei Spirit Oil Spill in South Korea. Land. DOI: Spill 10.3390/land10101089	Not meeting criterion *iii*
Appiah, S., Antwi-Asare, O.T., Agyire-Tettey, F.K, Abbey, E., Kuwornu, J.K.M., Cole, S. &Chimatiro, S.L. (2021). Livelihood Vulnerabilities Among Women in Small-Scale Fisheries in Ghana. The European Journal of Development Research, Palgrave Macmillan;European Association of Development Research and Training Institutes (EADI), 33,6,1596-1624.	Not meeting criterion *iii*
Lawless, S., Cohen, P.J., Mangubhai, S., Kleiber, D., & Morrison, T.H. (2021). Gender equality is diluted in commitments made to small-scale fisheries. *World Development,* 140, 105348. DOI:10.1016/j.worlddev.2020.105348	Not meeting criterion *i*
