# Supplementary material for: A synthesis of women’s participation in small-scale fisheries management: why women’s voices matter
Source: Rev Fish Biol Fish. 2023 Oct 18;34(1):43–63. doi: 10.1007/s11160-023-09806-2 (PMC10838824; doi:10.1007/s11160-023-09806-2)
Supplement: Supplementary file 2 — Supplementary file2 (PDF 222 kb) [file 11160_2023_9806_MOESM2_ESM.pdf]

SUPPLEMENTARY FILE 2

Article title: A Synthesis of Women's Participation in Small-Scale Fisheries Management: Why Women's Voices Matter

Journal name: *Reviews in Fish Biology and Fisheries*

Authors and Affiliations: Mouna CHAMBON[1], Sara MIÑARRO[1], Santiago ALVAREZ FERNANDEZ[1], Vincent PORCHER[1,2], Victoria REYES-GARCIA[1,3,4], Huran TONALLI DROUET[5], Patrizia ZIVERI[1,3,6]

[1] Institute of Environmental Science and Technology, Universitat Autònoma de Barcelona, (ICTA-UAB), 08193, Bellaterra, Barcelona, Spain.

[2] Unité mixte de recherche "Savoirs Environnement Sociétés"(SENS), Institut de recherche pour le développement (IRD) & Centre de coopération internationale en recherche agronomique pour le développement (Cirad), Montpellier, France.

[3] Institució Catalana de Recerca i Estudis Avançats (ICREA), Barcelona 08010, Spain.

[4] Dept. d'Antropologia Social i Cultural, Universitat Autònoma de Barcelona, 08193, Bellaterra, Barcelona, Spain.

[5] Université libre de Bruxelles, Avenue Franklin Roosevelt 50, 1050, Bruxelles, Belgium.

[6] Dept. de Biologia Animal, Biologia Vegetal i Ecologia, Universitat Autònoma de Barcelona, 08193, Bellaterra, Barcelona, Spain.

Corresponding author:
Mouna CHAMBON
Institute of Environmental Science and Technology, Universitat Autònoma de Barcelona (ICTA-UAB), 08193, Bellaterra, Barcelona, Spain
ORCID: 0000-0002-2260-5145; Tel : (+34) 6.67.58.05.87; E-mail :Mouna.Chambon@uab.cat

Online Resource 3- Tab. S3 Definition of variables used in the systematic literature review

Variable	***Definition***	***Format***
Women's participation in SSF management		
Women's participation level in SSF management	Women's participation level in SSF management processes	0= Excluded from management processes<br>1= Participation to some management activities but facing limitations to participate in decision-making processes<br>2= Active participation in management decision-making processes
Socio-cultural, environmental and economic impacts		
Impact Participation	The reported impact is related to the participation of women in management processes	0= No<br>1= Yes
Impact direction	The reported impact is presented as positive in the publication	0= No<br>1= Yes
Impact scale	Scale of the reported impact in the publication (select one only)	1= Socio-ecological system (SES) scale<br>2= Community scale<br>3= Individual scale
Impact category	Category of the reported impact in the publication (select one only)	1= Socio-cultural impact<br>2= Environmental impact<br>3= Economic impact
Impact subcategory	Subcategory of the reported impact in the publication (select one only)	1 = Change in the understanding of the gender dynamics within the SSF SES (Comprehensiveness)<br>2 = Change in the impact of management decisions on women (Gendered management impact)<br>3 = Change in the recognition of gendered ecological knowledge (Gendered ecological knowledge)<br>4 = Change in the compliance to management measures (Compliance)<br>5 = Change in the diversity of perspectives for SSF management (Diverse perspectives)<br>6 = Change in the long-term use of fisheries resources (Sustainable management)<br>7 = Change in human pressure on local ecosystems (Ecological pressure)<br>8 = Change in food security (Food security)<br>9 = Change in adaptive capacity (Adaptive capacity)<br>10 = Change in the social attributes of the community (Community social attributes)<br>11 = Change in the transmission of traditional knowledge (Cultural heritage)<br>12 = Change in community income (Community income)

		13 = Change in well-being (Well-being)<br>14 = Change in capacity building (Capacity building)<br>15= Change in women's empowerment and self-esteem (Empowerment)<br>16 = Change in gender roles (Gender roles)<br>17= Change in women's leisure time (Women's leisure time)<br>18= Change in women's income (Women's income)

Online Resource 4- Tab. S4 Adapted classification of the levels of women's participation in small-scale fishery management, after Agarwal's typology of women's participation in nature resource management (2001)

Corresponding level of participation	Agarwal's category	Characteristic features
**Excluded**	/	/
**Limited**	Nominal	Membership in the group
	Passive	Being informed of decision ex post facto; or attending meetings and listening in on decision-making, without speaking up
	Consultative	Being asked an opinion in specific matters without decisions
	Activity-specific	Being asked to (or volunteering to) undertake specific tasks
**Active**	Active	Expressing opinion, whether or not solicited or taking initiatives of other sorts
	Interactive (empowering)	Having voice and influence in the group's decisions
